# Building Trust: Strategies for Recruiting Underrepresented Populations in Research during the COVID-19 Pandemic

**DOI:** 10.21106/ijtmrph.446

**Published:** 2023-06-08

**Authors:** Leah K. Claus, Sara S. Jarvis

**Affiliations:** 1 Department of Biological Sciences, Northern Arizona University, 617 S Beaver, Flagstaff, AZ, 86011, USA

**Keywords:** Communities of Color, Minority, Research Recruitment, Barriers to Recruitment of Minorities, Successful Minority Recruitment, Cultural Barriers, Hispanic Community

## Abstract

The COVID-19 pandemic disproportionately affected racial and ethnic populations within the United States, creating a distinct set of circumstances that exacerbated barriers to research participation for underrepresented communities. This article aims to provide a rationale that validates the impact of the COVID-19 pandemic on these groups and suggests strategies for participant recruitment while sharing lessons learned from our own laboratory. By understanding the barriers that limit the recruitment of intended populations, researchers can implement culturally sensitive strategies and work towards a more inclusive body of literature with improved participation from underrepresented racial and ethnic populations.

## 1. Introduction

Historically, populations with the greatest burden of risk for disease have been underrepresented in research.^1^ As of 2019, communities of color constituted nearly 40% of the United States (US) population, yet their participation in clinical research ranged from 2–16%.^2^ This underrepresentation leaves gaps in the literature, which further contributes to health disparities for vulnerable populations.^3^ The COVID-19 pandemic created a unique set of challenges for research as almost 90% of NIH-funded non-commercial studies ceased to prioritize the safety of laboratory staff and participants.^4^ While this occurred, communities of color were disproportionately impacted by COVID-19.^5^

Native American populations experienced a death rate that was almost five times higher than national averages.^6^ Additionally, Hispanic/Latine populations experienced a death rate that was two times higher compared to Whites.^6^ The circumstances created by COVID-19 exacerbated barriers for underrepresented communities and further limited participation in research. Previous literature has suggested that individuals are less likely to participate in face-to-face research (not remote) during the COVID-19 pandemic.^4,7^ Researchers must acknowledge these barriers and employ strategies to improve representation across all racial and ethnic backgrounds. This article aims to 1) provide rationale validating the deleterious impact of COVID-19 on the recruitment of racial and ethnic diverse populations; 2) suggest strategies for authentic recruitment of these communities despite existing barriers; 3) describe our own laboratory practices and lessons learned.

## 2. Historical Recruitment of Underrepresented Populations

Despite mandates such as the Revitalization Act of 1993, enacted by the National Institutes of Health (NIH), underrepresentation in research persists for many communities, contributing to ongoing disparities.^2,8^ Increased racial and ethnic participation in research ensures findings can be applied to diverse populations, a need that is growing with the continued diversification in the US.^3^ This not only helps reduce the gap in the literature but could also answer warranted ethnic-specific questions that are currently unavailable due to insufficient data or sample sizes of racial and ethnic minorities.^9^

One of the largest barriers to participation in research studies is the individual’s perception of the scientific community. Many racial and ethnic populations experience a deep mistrust of scientific and medical communities. This is an adaptive response to chronic inequity, stemming from times of slavery and reinforced by more recent events such as the Tuskegee syphilis study and the Havasupai Trials.^10^ Both cases exploited vulnerable communities through a lack of informed consent and denial of treatment for a curable disease.^10^ These abuses of marginalized populations operated under the guise of scientific racism, or the manipulation of scientific practices to justify the dehumanization and enslavement of Black people.^11^ Contemporary examples of scientific racism continue to intensify mistrust by failing to acknowledge health disparities created by racism.^11^ These malpractices question the integrity of biomedical research, stressing the importance of recognizing the historical and contemporary roles that reinforce mistrust.^11^ Our intention should be to improve the trustworthiness of science research by properly interpreting findings within the context of structural racism.^11^

## 3. Additional Challenges from the COVID-19 Pandemic

The COVID-19 pandemic introduced unique challenges that further complicated the recruitment of underrepresented populations in research.^5^ The pandemic disproportionately affected these groups in a few ways. Comorbidities that are common in racial and ethnic communities, such as hypertension and diabetes mellitus, and an increased likelihood of living in densely populated neighborhoods contributed to a higher risk of COVID-19 exposure and infection.^5^ As a result, these populations were more hesitant about participating in research that would expose them further to an increased risk of COVID-19 infection.

Mistrust of the scientific and medical communities was also heightened by the COVID-19 pandemic.^12^ Media coverage often overlooked the underpinnings that led to disparities in COVID-19 infection and hospitalization, such as resource deficits, inadequate access to healthcare and testing, and pre-existing vaccine hesitancy prior to COVID-19.^12^ Despite efforts to include communities of color, there was ultimately a lack of diversity in COVID-19 vaccine clinical trials which reinforced vaccine hesitancy. This further fueled the existing mistrust of science within racial and ethnic underrepresented communities.^12^ Ultimately, these events increased the difficulty of recruitment for communities of color, as individuals lacked confidence in institutional research.

## 4. Discussion

Recruitment and retention of communities of color and other underrepresented populations in research have gained attention in recent years.^3^ Our ongoing NIH-funded research attempts to quantify insulin status and determine its relationship with endothelial function and blood pressure regulation in Hispanic and Latine individuals. This project began in the Fall of 2020 which limits our ability to assess the difficulty of recruitment prior to the COVID-19 pandemic. However, soon into the recruitment process, we realized we would need to adjust our strategies to specifically address the unique challenges posed by the COVID-19 pandemic, especially considering our target population’s higher risk of COVID-19 infection.^13,14^ We focused on addressing two key factors that impact recruitment success: safety and trust building. Within trust-building strategies, we highlight community-based participatory research and authentic community engagement.

Safety is a top priority for participants and staff. We implemented COVID-19 precautions, as recommended by the Centers for Disease Control and Infection (CDC), and communicated these measures to participants through consent documentation, verbal explanations, and our laboratory website. Additional personal protective equipment (PPE) was used while participating in tests (e.g. exercise), that raised respiration or depth to avoid the spread of a potential viral load. Taking these steps and providing these protocols helped subjects feel more comfortable participating in our research.

Building trust requires persistent efforts focused on community engagement and trust-building between research staff and the public.^10,14^ The community-based participatory research (CBPR) approach is one model that promotes partner-led recruitment by involving community and academic leaders in the research process.^1^ This model can add diversity to a research team to better represent the desired recruitment population while redirecting the dialogue to originate from a trusted member of the community. Ultimately, research needs to be conducted in a culturally respective manner with the understanding that building trust takes time and transparency in intent and methods is of the utmost importance.^15^ To help preserve the longevity of the relationship, findings should be returned to foster true partnership and sharing of data.^15^ We applied this model by working with a local entrepreneurial center that aims to provide opportunities for community members to gain an additional source of income. Building this relationship required our team to consistently honor our commitments to them and provide assistance as needed. After a foundation of trust was established, we were able to ask prominent leaders in the organization to help recruit and spread the word about our project. This shifted the perception of our laboratory from being outside of the community to being a part of the community, allowing our target population to feel more comfortable participating in our research.

Authentic community engagement can also help rebuild trust between the community at large and a research institution.^10^ By actively illustrating a willingness to help the community, we were able to foster specific community relationships that were needed to conduct research.^7^ We approached this through acts of service: attending local events, offering free blood pressure screenings, and providing bilingual pamphlets to help explain the importance of healthy blood pressure. We found that free blood pressure screenings were (and are) necessary and appreciated, especially with limited access to free or low-cost services since the COVID-19 pandemic. We took engagement a step further and volunteered in a few projects to restate our commitment to serving the community beyond research. Projects such as filling sandbags for flash-flooding (due to wildfire activity, which is common in the area) or distributing food at the local family food center taught the importance of listening to community needs. As a response, we started attending our local Diversity Council meetings to further practice active listening.

Despite additional challenges posed by the COVID-19 pandemic, our engagement efforts were positively received, evidenced by the increased enrollment of participants in our study. However, since the study is still ongoing, it is difficult to predict the ending impact and how that may be reflected in our study participants. Our laboratory intends to return to the community after the completion of the study and share significant findings so that future studies can build on the relationship already established. While our strategies were (and are) focused on recruiting the Hispanic/Latine population, we believe these lessons can be applied broadly to other communities. It is ultimately the responsibility of the researcher to understand the barriers limiting an intended recruitment population and implement culturally sensitive strategies to overcome them. By doing so, we can work towards a more inclusive body of literature and improve participation for underrepresented communities.

## Key Messages

- Although communities of color make up roughly 40% of the US population, only 2–16% of clinical research reflects these communities. This represents a vast need to increase participation in racial and ethnic communities.
- The COVID-19 pandemic created a unique set of circumstances that worsened the barriers that prevent and discourage clinical research participation among underrepresented populations.
- Our laboratory addressed strategies to promote the feeling of safety among participants while also working to build trust with the Hispanic/Latine population in our town. This article shares lessons learned that can be broadly applied to other underrepresented populations.
- It is ultimately the responsibility of the researcher(s) to understand the barriers that discourage research participation for their intended recruitment population. Once these barriers are understood, culturally sensitive strategies can be used to overcome and increase representation in the project.

## References

[1] Horowitz CR, Brenner BL, Lachapelle S, Amara DA, Arniella G. Effective recruitment of minority populations through community-led strategies. Am J Prev Med. 2009;37(6, Supplement 1):S195–S200. doi:10.1016/j.amepre.2009.08.006 19896019 PMC2810631

[2] Oh SS, Galanter J, Thakur N, Diversity in clinical and biomedical research: a promise yet to be fulfilled. PLoS Med. 2015;12(12):e1001918. doi:10.1371/journal.pmed.1001918 26671224 PMC4679830

[3] Yancey AK, Ortega AN, Kumanyika SK. Effective Recruitment and Retention of Minority Research Participants. Annu Rev Public Health. 2006;27(1):1–28. doi:10.1146/annurev.publhealth.27.021405.102113 16533107

[4] Mirza M, Siebert S, Pratt A, Impact of the COVID-19 pandemic on recruitment to clinical research studies in rheumatology. Musculoskeletal Care. 2022;20(1):209–213. doi:10.1002/msc.1561 33938621 PMC8242596

[5] Alcendor DJ. Racial disparities-associated COVID-19 mortality among minority populations in the US. J Clin Med. 2020;9(8):2442. doi:10.3390/jcm9082442 32751633 PMC7466083

[6] Macias Gil R, Marcelin JR, Zuniga-Blanco B, Marquez C, Mathew T, Piggott DA. COVID-19 pandemic: disparate health impact on the Hispanic/Latinx population in the United States. J Infect Dis. 2020;222(10):1592–1595. doi:10.1093/infdis/jiaa474 32729903 PMC7454709

[7] Ojikutu BO, Stephenson KE, Mayer KH, Emmons KM. Building trust in COVID-19 vaccines and beyond through authentic community investment. Am J Public Health. 2021;111(3):366–368. doi:10.2105/AJPH.2020.306087 33301352 PMC7893367

[8] Chastain DB, Osae SP, Henao-Martínez AF, Franco-Paredes C, Chastain JS, Young HN. Racial disproportionality in Covid clinical trials. N Engl J Med. 2020;383(9):e59. doi:10.1056/NEJMp2021971 32780573

[9] Elliott MN, McCaffrey DF, Finch BK, Improving disparity estimates for rare racial/ethnic groups with trend estimation and Kalman filtering: an application to the National Health Interview Survey. Health Serv Res. 2009;44(5 Pt 1):1622–1639. doi:10.1111/j.1475-6773.2009.01000.x 19656232 PMC2754551

[10] Ojikutu BO, Bogart LM, Dong L. Mistrust, empowerment, and structural change: lessons we should be learning from COVID-19. Am J Public Health. 2022;112(3):401–404. doi:10.2105/AJPH.2021.306604 35196050 PMC8887168

[11] Opara IN, Riddle-Jones L, Allen N. Modern Day Drapetomania: Calling Out Scientific Racism. Gen Intern Med. 2022;37(1):225–226. doi:10.1007/s11606-021-07163-z PMC8513734 34647230

[12] Bogart LM, Ojikutu BO, Tyagi K, COVID-19 related medical mistrust, health impacts, and potential vaccine hesitancy among Black Americans living with HIV. J Acquir Immune Defic Syndr. 2021;86(2):200–207. doi:10.1097/QAI.0000000000002570 33196555 PMC7808278

[13] Arizona: all race & ethnicity data. The COVID Tracking Project. Published March 2022. Accessed April 4, 2022. https://covidtracking.com/data/state/arizona/race-ethnicity

[14] Best AL, Fletcher FE, Kadono M, Warren RC. Institutional distrust among African Americans and building trustworthiness in the COVID-19 response: implications for ethical public health practice. J Health Care Poor Underserved. 2021;32(1):90–98. doi:10.1353/hpu.2021.0010 33678683 PMC7988507

[15] Credo J, Ingram JC. Perspective developing successful collaborative research partnerships with AI/AN communities. Int J Environ Res Public Health. 2021;18(17):9089. doi:10.3390/ijerph18179089 34501677 PMC8430766

